# Aqueous Chlorination of D-Limonene

**DOI:** 10.3390/molecules27092988

**Published:** 2022-05-06

**Authors:** Albert T. Lebedev, Elena A. Detenchuk, Tomas B. Latkin, Mojca Bavcon Kralj, Polonca Trebše

**Affiliations:** 1 Organic Chemistry Department, Lomonosov Moscow State University, Leninskie Gory 1/3, 119991 Moscow, Russia; helen-detenchuk@mail.ru; 2 MASSECO d.o.o., 6230 Postojna, Slovenia; 3 Core Facility Arktika, Northern Arctic Federal University, 163002 Arkhangelsk, Russia; tomdamnnorth@gmail.com; 4 Faculty of Health Sciences, University of Ljubljana, 1000 Ljubljana, Slovenia; mojca.kralj@zf.uni-lj.si (M.B.K.); polonca.trebse@zf.uni-lj.si (P.T.)

**Keywords:** limonene, aqueous chlorination, GC-HRMS, haloforms, electrophilic addition, disinfection by-products

## Abstract

Limonene (1-methyl-4-(1-methylethenyl)-cyclohexene) is one of the most widespread monocyclic terpenes, being both a natural and industrial compound. It is widely present in the environment, including in water supplies. Therefore, it may be subjected to aqueous chlorination at water treatment stations during drinking water preparation. Besides, being a component of numerous body care and cosmetic products, it may present at high levels in swimming pool waters and could also be subjected to aqueous chlorination. Laboratory experiments with aqueous chlorination of D-limonene demonstrated the prevalence of the conjugated electrophilic addition of HOCl molecule to the double bonds of the parent molecule as the primary reaction. The reaction obeys the Markovnikov rule, as the levels of the corresponding products were higher than those of the alternative ones. Fragmentation pattern in conditions of electron ionization enabled the assigning of the structures for four primary products. The major products of the chlorination are formed by the addition of two HOCl molecules to limonene. The reactions of electrophilic addition are usually accompanied by the reactions of elimination. Thus, the loss of water molecules from the products of various generations results in the reproduction of the double bond, which immediately reacts further. Thus, a cascade of addition-elimination reactions brings the most various isomeric polychlorinated species. At a ratio of limonene/active chlorine higher than 1:10, the final products of aqueous chlorination (haloforms) start forming, while brominated haloforms represent a notable portion of these products due to the presence of bromine impurities in the used NaOCl. It is worth mentioning that the bulk products of aqueous chlorination are less toxic in the bioluminescence test on *V. fischeri* than the parent limonene.

## 1. Introduction

Disinfection of water is essential in order to deactivate pathogenic microorganisms and prevent different infectious illnesses. Chlorination, as the first disinfection approach for water, was introduced in 1902 in Belgium. Since then, it has been known as an efficient method for municipal water disinfection. Over the years, several other disinfection methods were developed (among others, ultraviolet irradiation and ozonation, separately or in different combination, were applied and are known as advanced oxidations processes (AOP)). They include beside photolysis (ultraviolet (UV) or vacuum-UV), hydrogen peroxide (H_2_O_2_)-based techniques, ozone (O_3_)-based techniques, and heterogeneous photocatalysis [1]. The advantage of chlorination lies in inactivation of pathogens, its cheapness, stability in distribution systems, and simplicity of use. However, one definite disadvantage involves the fact that chlorine or chlorine-based compounds react with organic matter and other human outputs, forming different disinfection by-products (DBPs).

Over 30 years, we have been studying DBPs in drinking water forming due to chlorination (ozonation) of natural and anthropogenic organic compounds [2,3,4,5,6,7,8,9,10]. Numerous DBP including novel ones were detected in these studies, whereas there is no toxicological information about the vast majority of these products. Nevertheless, these products may be rather hazardous for humans, belonging to the classes of haloforms, halogenated phenols, aldehydes, acids, acetophenones, or polychlorinated aromatic species.

Emerging contaminants, such as personal care products, pharmaceuticals, or different industrial additives, are a group of unregulated compounds, causing possible harmful effect on humans and other non-target organisms when present in the water [11]. Nowadays, more than 700 emerging contaminants, their metabolites, and transformation products, are present in the European aquatic environment (www.norman-network.net, Accessed on 11 November 2021). They are currently not included into (inter)national routine monitoring programs and their fate, behavior and ecotoxicological effects are often not well understood.

D-Limonene is produced industrially since 1995, being used primarily as an additive of taste or odor in food and drinks, numerous cosmetics, pharmaceutical, and cleaning products [12,13,14,15]. Nowadays, it is often used as a solvent in resins production, as a wetting and dispersing agent, as well as a repellent [16,17]. It was shown [18] that 95% of 280 studied perfumery products and 69% of 150 deodorants contained limonene. The traces of limonene are often detected in human bodies, while the highest levels of limonene are discovered in the region of face and neck (0.25 mg/sm^2^/day). These levels were mainly due to the citrus’s essential oils.

**Figure 1:**
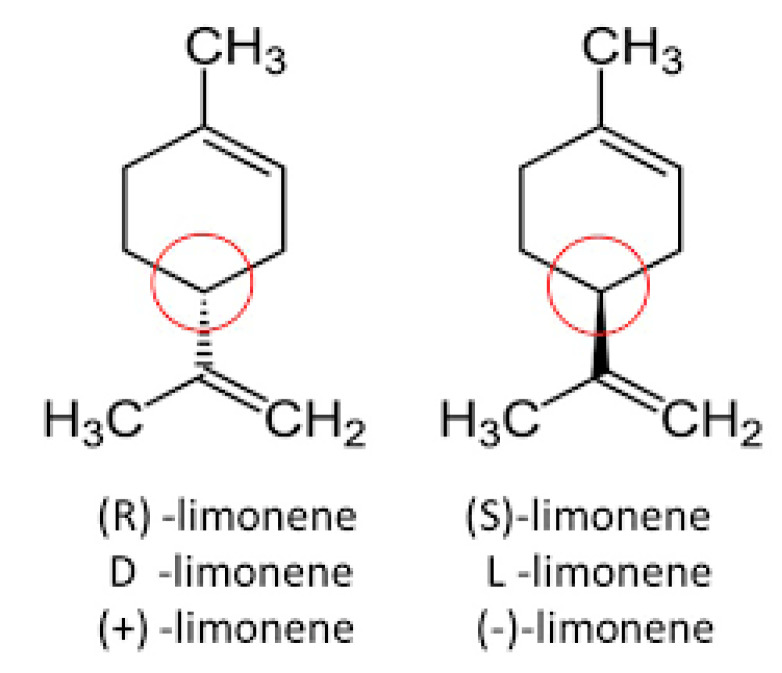
The structural formulas of isomeric D- and L-limonenes.

Limonene (1-methyl-4-(1-methylethenyl)-cyclohexene) is one of the most widespread monocyclic terpenes produced by over 300 plants all over the world [19,20]. Possessing an asymmetric carbon atom, it is represented in nature by two optical isomers: L-limonene and D-limonene (Figure 1). The isomers have different smells. L-limonene (CAS 5989-54-8) smells similar to pine or turpentine, while D-limonene (CAS 5989-27-5) demonstrates a pleasant aroma of oranges, being the main component of the majority of the citrus oils [17,21]. It is also present in oils of cumin, neroli, bergamot, caraway [22]. Often limonene is present as a mixture of both D,L-isomers (CAS 138-86-3) with a trivial name of dipentene.

Among various flavoring ingredients, D-limonene accounts for higher usage, with 92% of total annual volume in the USA [23]. D-limonene is registered as a common safe compound (GRAS) in the Codex of Federal Regulations (CFR) for the synthetic aromatizers [24]. Being rather volatile, D-limonene is often present in various interior spaces [25,26,27,28,29,30,31,32] easily reacting with ozone and forming various toxic products [33,34] due to two double bonds. These products are quite complex mixtures of highly volatile compounds [35,36] and secondary organic aerosols [34,37,38,39] in the form of fine and ultrafine particles (UFP) [36,40,41,42]. The identified products of limonene oxidation include aldehydes (formaldehyde and acrolein), formic and acetic acid, various alcohols and terpene derivatives [43], carbonyl compounds [44], hydroperoxides of limonene, R-carvone, and limonene oxide as well as radical species [45,46]. These radicals may bring certain negative consequences in pulmonary tract [39]. Airborne exposure of mice with limonene (52 ppm) and its oxidation products due to the addition of ozone (0.5–3.9 ppm) for one hour results in irritation of the eyes, nose, and skin progressing during 10 days [33].

Despite the application of D-limonene as an additive of taste enhancing in food industry, it was shown that it might induce certain toxic effects, especially in the case of rats [47,48]. LD50 values for R-(+)-limonene vary from 0.125 g/Kg for rats (intravenous) to >55 g/Kg for rabbits (dermal) [49]. The risks related to the application of limonene as well as its toxicology and pharmacokinetics were reported in [49]. Human exposure with D-limonene involves food and drinks consumption, application of cosmetic products, and even inhalation as it is often present in the air. Oxidation products of limonene possess higher cannibalizing potential than the parent compound [49]. R-(+)-limonene demonstrated negative results in the Ames test, proving the absence of any mutagenic activity [50].

R-(+)-limonene easily evaporates from water, wet and dry soil, although its absorption to the soil ingredients may slow down that process [51]. Half-life period of the presence of limonene in the model river water before evaporation (depth 1 m, flow rate 1 m/s, and wind speed 3 m/s) is 3.4 h [51,52] Its bioaccumulation in fish and other aquatic species was also reported [51,52]. Limonene poorly hydrolyses, although its biodegradation takes place both in aerobic and anaerobic conditions. Limonene is biodegradable in soil and photodegradable is wet air under the solar irradiation [52].

Due to its presence in numerous products and its natural sources, limonene may be detected almost in any environmental samples. Since 2010, we have regularly detected that compound during monitoring of surface water and precipitations in Moscow, e.g., Moscow snow [53,54] and rain [55], Arctic snow [56], and French cloud water [57] samples. Appearing at the water treatment stations producing drinking water, limonene as a very reactive compound easily interacts with active chlorine forming various disinfection by-products (DBP) [58].

The present study sheds light upon the aqueous chlorination of D-limonene, which may take place during drinking water preparation or in swimming pool water. In order to identify primary as well as secondary products, concentrations higher than those relevant for aquatic environment have been applied in chlorination experiments. Gas chromatography with high resolution mass spectrometry (GC-HRMS) was used to identify and estimate the levels of volatile and semi volatile disinfection by-products. The inhibition of *V. fischeri* luminescence test was used to estimate toxicity of the forming DBPs.

## 2. Materials and Methods

*Reagents.* 1-methyl-4-prop-1-en-2-ylcyclohexene (D-limonene, ≥99.0% sum of enantiomers, GC) was obtained from Sigma–Aldrich (Darmstadt, Germany). Sodium hypochlorite and dichloromethane (extra pure) were purchased from Neva Reactiv, Saint-Petersburg, Russia. Sodium thiosulfate (standard-title) was obtained from Uralhiminvest, Russia. Sodium Sulfate anhydrous (Reag. USP) for analysis and sodium sulfite were obtained from Panreact, Spain. Sodium chloride (especially pure) was obtained from Component-reactiv, Russia. Deuterated standards (99.8%) were obtained from Deutero GmbH, Germany. Milli-Q water from a Milli-Q Plus water purification system (Millipore, Merck KGaA, Darmstadt, Germany) was used.

*Aqueous chlorination.* To prepare reaction mixture limonene: active chlorine 2:1 limonene solution (95 µL, concentration 0.05 g/L) and 2.95 µL of sodium hypochlorite solution with active chlorine concentration 0.025 g/L were added to 10 ml of phosphate buffer (pH 7). Six samples and one blank (without limonene) were prepared that way. The reaction mixtures were kept in the dark at the room temperature for 5, 10, 15, 30, 45, and 60 minutes. An excess of sodium sulfite was added to delete the remaining active chlorine. An aliquot (5 mL) was transferred into a vial for the headspace analysis. The vial was tapped after adding 2 g NaCl. Then, 5 mL of dichloromethane was added to the remaining portion of the reaction mixture to extract the reaction products. Perdeuterated PAH were added as internal standards. Similarly, reactions of limonene with active chlorine were launched at the limonene/active chlorine ratio 5:1, 1:1, 1:2, 1:5, 1:10, and 1:20. The levels of limonene introduced into the reaction were intentionally higher than in natural water reservoirs in order to define as many transformation products as possible.

*GC-HRMS analysis.* Gas Chromatograph Agilent 7890A (Agilent Technologies, Palo Alto, CA, USA) with time-of-flight mass spectrometer Pegasus^®^ GC-HRT (LECO Corporation, Saint Joseph, MI, USA was used. The data were acquired using 15 full (15–900 *m*/*z* range) spectra per second with resolving power 25,000. Chromatographic separation was achieved using an Rxi-5MS (30 m × 0.25 mm (id) × 0.25 µm, Restek Corporation, Bellefonte, PA) column with a constant helium flow of 1.2 mL/min. All injection volumes were 1 µL, with split 1:10. The injector and the transfer line temperatures were set at 280 °C and 320 °C, respectively. The GC oven program was as follows: isothermal at 50 °C (1 min), then 5 °C/min ramping to 300 °C and 9 min isothermal hold at that temperature. Ion source temperature −200 °C, ionization energy—70eV. Perdeuteronaphthalene and perdeuterophenanthrene were used as internal standards for quantification. Response factor was calculated only for the parent limonene. For all of the detected products response factor was taken as 1.

Haloforms were identified and quantified using headspace analysis with the same instrument in GC/MS mode. The 20 mL vial with 5 mL of the reaction mixtures after chlorination was thermostated at 80 C during 30 min with 600 rpm agitation. 1 mL of the headspace sample was injected into the instrument. Chromatographic separation was carried out with Rxi-5SilMS 30 m × 0.25 mm (id) × 0.25 µm (df) (Restek, Bellefonte, PA, USA). The injector temperature was 250 °C. The carrier gas flow was 1.0 mL/min. The sample was injected in the flow split mode 1:10. The GC oven program was as follows: a 2-min isothermal hold at 40 °C, and then ramping at 5 °C min^−1^ to 250 °C followed by a 2-min isothermal hold at 250 °C. Mass spectra for all GC runs (1D mode) were acquired at a rate of 15 spectra per second in the mass range of *m*/*z* 29–500. The mass spectrometer was operated in a high-resolution mode with a resolution of 25,000 or more. The temperature of the ion source was 250 °C. The quantification was carried out using preliminary calibration with standard mixture of volatile pollutants (US EPA Method 8260, Restek, Bellefonte, PA, USA).

*Toxicity measurements.* The toxicity assessment of limonene aquatic solution within chlorination experiments has been performed using liquid dried luminescent bacteria *V. fischeri* with system LUMIStox, Dr. Lange according to ISO 11348-2 (International Organization for Standardization, 1998). The toxicity endpoint was determined as reduced luminescence emission after 30 min incubation of a selected chlorination mixture (limonene/active chlorine ratio 5:1, 2:1, 1:1, 1:2, 1:5, 1:20) as it was described in the section Aqueous chlorination. Details about the complete procedure have been published elsewhere [7].

## 3. Results and Discussion

**Scheme 1:**
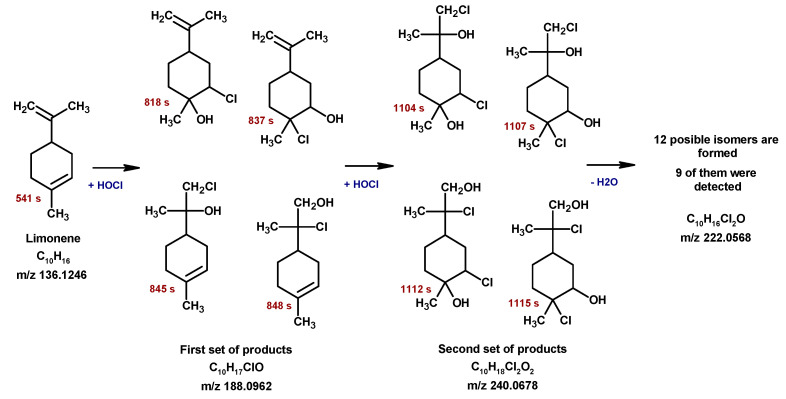
Main pathways of limonene transformation in conditions of aqueous chlorination with sodium hypochlorite.

**Figure 2:**
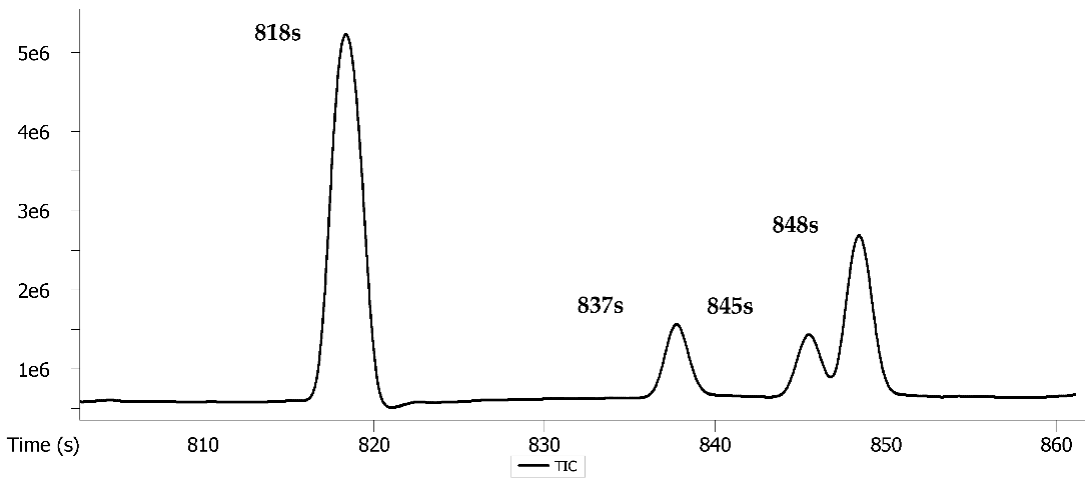
TIC chromatogram segment demonstrating four primary products of limonene aqueous chlorination. All four numbers shifted away from the tops of the corresponding peaks. I do not know how to place them correctly.

**Figure 3:**
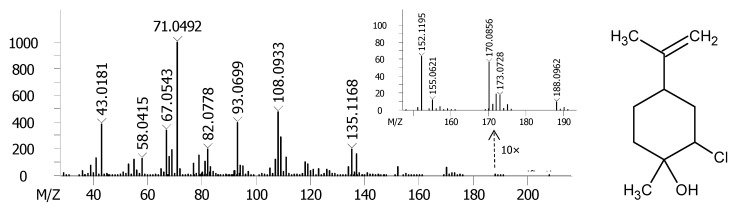
Mass spectrum and formula of 1-methyl-2-chloro-4 (1-methyletenyl)cyclohexanol with RT 818 s.

**Figure 4:**
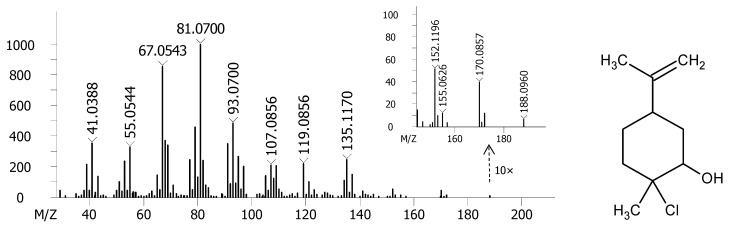
Mass spectrum and formula of 2-chloro-2-methyl-5-(1-methyletenyl)cyclohexanol with RT 837 s.

**Figure 5:**
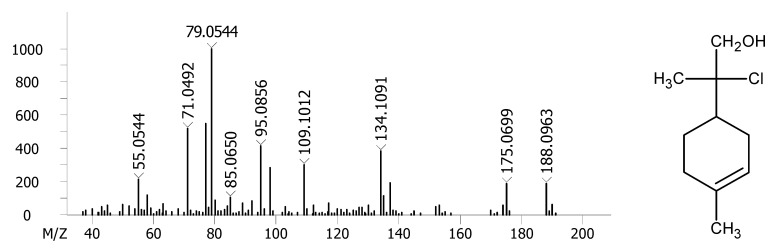
Mass spectrum and formula of 1-methyl-4-(2-chloro-1-hydroxyprop-2-yl)cyclohexene with RT 845s.

**Figure 6:**
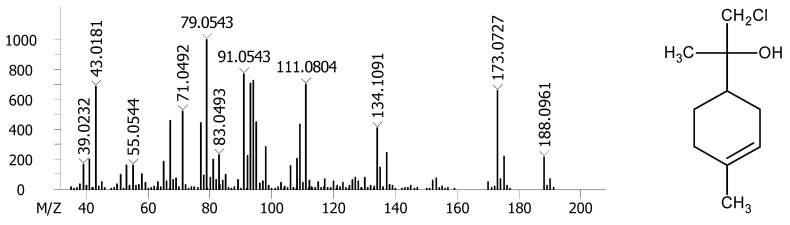
Mass spectrum and formula of 1-methyl-4-(1-chloro-2-hydroxyprop-2-yl)cyclohexene with RT 848s.

The primary reaction of the aqueous chlorination of limonene involves electrophilic addition to the double bond (Scheme 1). Both double bonds of the molecule are available for that reaction. Since the level of chloride anions is incomparably lower than that of the water molecules, the mechanism of conjugated addition dominates. The chloronium ion forming at the first step reacts mainly with water, resulting in formation of four primary products with general formula C_10_H_17_ClO, two of which (**818s** and **848s**) follow Markovnikov rule [59] and two (**837s** and **845s**) violate the rule (Figure 2). Since the double bond in the ring has three alkyl substitutes, its nucleophilicity is higher than that of the isopropylene group. As a result, the yields of the corresponding products are higher. Alternative dichloro derivatives were detected at trace levels only in the case of the highest ratio limonene/active chlorine. Unfortunately, not a single product was characterized earlier by its mass-spectrum. Therefore, it was not possible to use NIST or WILEY libraries. However, a number of the corresponding alcohols, where chlorine is substituted for hydrogen, allowed deriving some valuable conclusions on the basis of the unique characteristic fragment ions. Another issue worth mentioning involves the fact that the addition of HOCl to the double bond in the cycle creates two additional chiral centers. Therefore, diastereomeric products should be formed in the reaction. Their physicochemical properties (retention times) seem to be rather close, preventing their separation at the chromatographic column and leading only to four separate peaks (Figure 2). Mass spectra (Figure 3, Figure 4, Figure 5 and Figure 6) represent superposition of the spectra of the corresponding diastereomers. The number of diastereomers becomes even greater at the advanced stages of chlorination (Scheme 1).

The major chromatographic peak (**818s**) corresponds to the conjugated electrophilic addition into the cycle according to the Markovnikov rule. Its molecular ion of *m*/*z* 188.0962 is of low abundance. The primary fragment ions are [M−CH_3_]^+^ and [M−H_2_O]^+^ (Figure 3). The base peak in the mass spectrum of *m*/*z* 71.0491 may be represented by the formula CH_2_=CH-CCH_3_=OH^+^ being formed through the classic reaction of fragmentation of cyclohexanol derivatives [60]. Alternative fragment ion of *m*/*z* 105.0107-C_4_H_6_ClO, forming by the primary cleavage of C1-C6 bond is also present in the spectrum.

A minor peak with RT **837s** (Figure 4) forms due to reaction in the cycle against Markovnikov rule. Fragmentation of cyclohexanol ring leads to the formation of the characteristic ion C_6_H_7_O of *m*/*z* 95.0492. Molecular ion of *m*/*z* 188.0962 is hardly visible, while [M−CH_3_]^+^ ion is absent.

The products of HOCl addition into the side chain demonstrate higher intensities of their molecular ions. The minor peak **845s** is due to anti-Markovnikov product. Its molecular ion and [M−CH_3_]^+^ fragment ion are quite abundant (Figure 5). Retro-Diels-Alder reaction results in formation of two fragment ions of low abundance of *m*/*z* 68.0621 and 120.0336.

As expected, Markovnikov product (**848s**) into the side chain is more pronounced being the second among the primary products of limonene in Figure 2. Its molecular ion (Figure 6) is rather abundant. Retro-Diels-Alder reaction again results in formation of two fragment ions of *m*/*z* 68.0621 and 120.0336.

**Figure 7:**
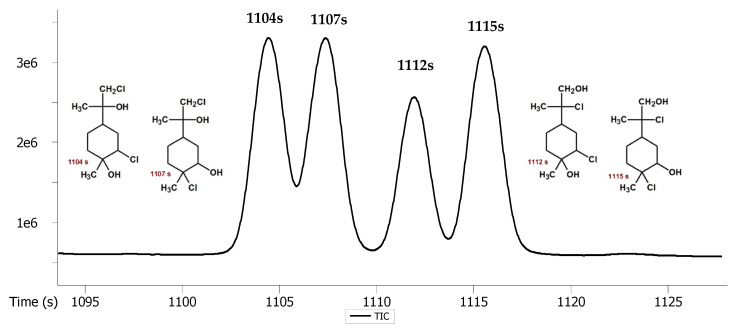
TIC chromatogram segment demonstrating four secondary products of limonene aqueous chlorination.

Conjugated electrophilic addition to the second double bond of the primary products results in formation of four secondary products with the general formula C_10_H_18_Cl_2_O_2_, with all possible combinations of chlorine and hydroxyl moieties in the molecules. These are compounds with retention times **1104s**, **1107s**, **1112s**, and **1115s** (Figure 7). Formation of these products is favorable (Table 1) while their levels are higher than that of any other products. Mass spectra of the products are presented in Supplementary Materials (Figures S1–S4). It is quite difficult to assign the spectra to the correct structures. Only the loss of CH_2_Cl radical from their molecular ions allows defining compounds 1104s and 1112s as Markovnikov products into the side chain of limonene. Anyway, an assessment has been tentatively carried out. 

**Figure 8:**
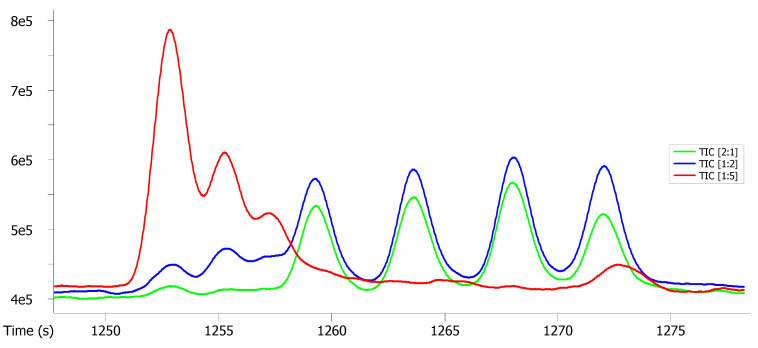
TIC chromatogram of the polychlorinated products of limonene aqueous chlorination depending on the ratio limonene/active chlorine: [2:1], [1:2], and [1:5].

Electrophilic addition reactions during aqueous chlorination of limonene are accompanied by the elimination processes. The latter involves the loss of water molecule forming another array of isomeric products with general formula C_10_H_16_Cl_2_O. Theoretically, it is possible to form 12 isomers. However, we managed to detect only nine of them. It is likely that some of them have very similar retention times and coelute or being less favorable to be formed they are present in trace levels. It is impossible to elucidate their unique structures reliably. Anyway, it is not strictly necessary as their toxicological properties should be quite similar. The latter group of products can continue react with active chlorine with formation of products of new generations. Actually, consecutive reactions of addition and elimination may result in the formation of highly halogenated species, which usually appear to be more toxic. Since more and more isomeric products appear at each new step, their levels become lower and lower. Molecular ions of the corresponding polychlorinated compounds are not stable, but their *m*/*z* values may be calculated based on the losses of water molecules and chlorine atoms. For example, ion of *m*/*z* 239.0601 (C_10_H_17_Cl_2_O_2_)—is the primary fragment ion of the trichlorinated product C_10_H_17_Cl_3_O_2_, formed due to the loss of chlorine atom. These polychlorinated compounds arise at the ratio limonene:active chlorine 1:1, 1:2 and 1:5. They have general formula C_10_H_x_Cl_y_O_z_ (x = [14,15,16,17]; y > 2; z = [1,2]). C_10_H_17_Cl_3_O_2_, C_10_H_16_Cl_4_O_2_, C_10_H_15_Cl_3_O, C_10_H_14_Cl_4_O. Figure 8 illustrates a short chromatographic segment demonstrating formation of such compounds, depending on the ratio limonene:active chlorine.

**Figure 9:**
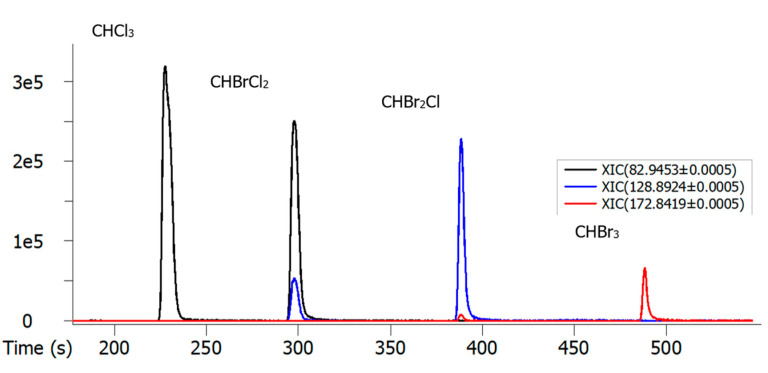
Ion chromatograms based on the characteristic ions of haloforms formed in aqueous chlorination of limonene at the ratio limonene/active chlorine 1:20. XIC means extracted ion chromatograms.

It is well known that the final products of transformation of organic compounds in aqueous chlorination are haloforms [11,61,62,63,64,65]. Haloforms are identified and quantified at drinking water treatment stations all over the world. Moreover, their toxicities are well known. Therefore, it was important to check the possibility of formation of these hazardous compounds from limonene. Headspace analysis was used for that purpose [66,67]. The samples with the ratio limonene/active chlorine: [5:1], [2:1], [1:1], [1:2], [1:5] as well as the corresponding blanks did not contain any representatives of that class. However, at the ratio [1:10] and [1:20] all possible chlorobromomethanes were reliably detected and quantified using the available standards (Figure 9). It is interesting that the major product among haloforms appeared to be bromodichloromethane (7.43 ppm) followed by chloroform (4.15 ppm), dibromochloromethane (1.91 ppm), and finally bromoform (0.15 ppm). Formation of the corresponding organobromines may be rationalized by the presence of hypobromite in the sodium hypochlorite reagent and its significantly higher reactivity comparing to the latter [10,68].

The principal pathway of aqueous chlorination of limonene is represented in Scheme 1, while the levels of the detected products at various ratios limonene/active chlorine are summarized in Table 1.

### Toxicity Assessment

**Figure 10:**
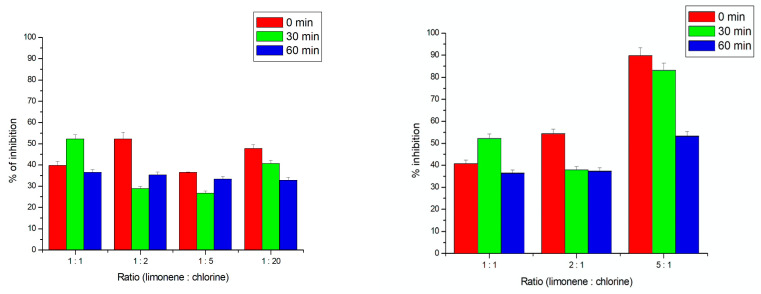
Bioluminescence inhibition of *V. fischeri* for selected chlorination mixtures of limonene/active chlorine ratio 1:1, 1:2, 1:5, 1:20 (**left**) and 1:1, 2:1, 5:1 (**right**) in buffer solution at 0, 30, and 60 minutes.

The results of toxicity measurements are presented in Figure 10. Various amounts of chlorine were added to the samples of limonene and prepared in buffer solution in order to reach limonene/active chlorine ratios 1:1, 1:2, 1:5, and 1:20, respectively. The inhibition of *V. fischeri* luminescence for selected chlorination mixtures do not vary much (not among different ratios (up to 15%) or with time of exposure (up to 20%)). We might say they are comparable and lead us to the possible assumption that the toxicity of chlorinated products formed in these cases is comparable (Figure 10—left). It should be stressed that only haloforms have been quantified. Single compound toxicity assessment would give us proper information. Since we lack standards, such assessment was not possible to perform.

On the other hand, in the case of samples with limonene/active chlorine ratio 1:1, 2:1, and 5:1, it was obvious that the luminescence inhibition depends on the amount of limonene in the mixture and not on the amount of chlorine added (Figure 10—right). Actually, in the case of mixture with ratios of limonene:chlorine 1:1 and 2:1, we may observe increased inhibition already at the beginning, before the addition of chlorine, but after 60 min of exposure, there is no difference in bioluminescence inhibition between both samples. This could be explained with the formation of different chlorinated products and their quantities. Comparing these results with that for the sample 5:1, the increased inhihition of luminescence before the addition of chlorine as well as after 30 min of exposure, when it reached more than 80%, is clearly visible. After 60 min of exposure, the inhibition was higher in comparison with the inhibition of samples with limonene:chlorine ratio 1:1 and 2:1, but for only 10%. In that case we may assume that chlorinated products could be less toxic for *V. Fischeri* than parent limonene taking into account that the average relative standard deviation of Lumistox assay was 5%.

## 4. Conclusions

Aqueous chlorination of D-limonene starts with the conjugated electrophilic addition of HOCl molecule to the double bonds of the parent molecule. The fragmentation pattern in conditions of electron ionization allowed for assigning the structures for four primary products. The levels of both Markovnikov products were higher than that of anti-Markovnikov ones.

The major products of the chlorination are formed by addition of two HOCl molecules to limonene. Since the reactions of electrophilic addition are accompanied by the reactions of elimination (water molecule), double bond in the substrate is reproduced at new and new stages, making possible the formation of the most various isomeric polychlorinated species.

At the ratio limonene/active chlorine higher than 1:10 haloforms start forming as the final products of aqueous chlorination. It is worth mentioning that brominated haloforms represented a notable portion of these products due to the presence of bromine impurities in the used NaOCl [10,68].

Toxicities of the products appeared to be not high at all. Actually, the products are less toxic in the bioluminescence test on *V. fischeri* than the parent limonene.

## Figures and Tables

**Table 1:** Main products of limonene transformation in conditions of aqueous chlorination with sodium hypochlorite.

No	Compound/Formula	RT, s	Quantity at the Ratio of Limonene:Chlorine, µg						
			Blank	10:1	5:1	2:1	1:1	1:2	1:5
1	D-Limonene C_10_H_16_	541	17.7	16.9	10.6	0.9	1.5	7.3	1.3
2	Primary products C_10_H_17_ClO Mm 188	818		5.1	12.2	14.8	0.6	12.5	0.4
3		837		0.3	1.3	0.01		1.0	
4		845		0.1	0.3	0.4	4.5	0.4	0.6
5		848		0.4	0.9	1.5	18.6	1.4	2.0
6	Elimination products C_10_H_16_Cl_2_O Mm 222	989				0.1	0.3	0.03	0.3
7		991				0.1	0.6	0.2	0.1
8		996				0.2	1.0	0.4	1.1
9		1015		0.1	0.2	3.3	15.0	2.4	8.3
10		1017		0.8	0.1	2.3	15.4	0.9	16.3
11		1021		0.01	0.5	0.01	0.2	0.3	0.1
12		1023		0.03	0.4	0.01	0.2	0.2	0.7
13		1030		0.04	0.1	0.3	0.9	0.4	1.0
14		1037			0.01	0.01	0.1	0.04	0.1
18	Secondary products C_10_H_18_Cl_2_O_2_ Mm 240	1104		0.6	0.9	1.7	7.7	4.2	5.4
19		1107		0.4	0.5	3.2	8.4	3.4	9.0
20		1112		0.6	1.0	3.0	8.4	4.2	8.9
21		1115		0.9	1.3	3.7	9.3	5.2	9.9
22	Polychlorinated products C_10_H_x_Cl_y_O_z_ (x = [14,15,16,17]; y > 2; z = [1,2])	1229					0.3	0.03	0.3
23		1231					0.1	0.01	0.1
24		1233					0.01	0.01	0.3
25		1235			0.2	0.2	0.1	0.2	
26		1239			0.2	0.3	0.2	0.4	
27		1248			0.3	0.4	0.2	0.5	
28		1252			0.1	0.3	0.2	0.3	0.03

## Supplementary Materials

The following supporting information can be downloaded at https://www.mdpi.com/article/10.3390/molecules27092988/s1, Figure S1: Mass spectrum and formula of 1-methyl-2-chloro-4(2-chloro-1-hydrohypropyl-2)cyclohexanol with RT 1104 s; Figure S2: Mass spectrum and formula of 2-chloro-2-methyl-4(2-chloro-1-hydrohypropyl-2)cyclohexanol with RT 1107 s; Figure S3: Mass spectrum and formula of 1-methyl-2-chloro-4(1-chloro2-hydroxy-propyl-2)cyclohexanol with RT 1112 s; Figure S4 Mass spectrum and formula of 2-chloro-2-methyl-4-(1-chloro2-hydroxy-propyl-2)cyclohexanol with RT 1115 s.

## Sample Availability

Samples of the compounds are not available from the authors.
