# Supplementary material for: Aqueous Chlorination of D-Limonene
Source: Molecules. 2022 May 6;27(9):2988. doi: 10.3390/molecules27092988 (PMC9099452; doi:10.3390/molecules27092988)
Supplement: Supplementary file 1 [file molecules-27-02988-s001.zip › molecules-1694598-supplementary.pdf]

molecules

MDPI

Supplementary Materials

Aqueous Chlorination of D-limonene

Albert T. Lebedev [1,2,*], Elena A. Detenchuk [1], Tomas B. Latkin [3], Mojca Bavcon Kralj, [4] and Polonca Trebše [4]

1 Organic Chemistry Department, Lomonosov Moscow State University, Leninskie Gory 1/3, 119991 Moscow, Russia; a.lebedev@org.chem.msu.ru (L.A.T); helen-detenchuk@mail.ru (D.E.A.)
2 MASSECO d.o.o., Postojna, 6230 Slovenia; mocehops@yandex.ru
3 Core Facility Arkrika; Northern Arctic Federal University, 163002, Arkhangelsk, Russia; tomdamn-north@gmail.com
4 Faculty of Health Sciences, University of Ljubljana, 1000 Ljubljana, Slovenia; mojca.kralj@zf.uni-lj.si (M.B.K.); polonca.trebse@zf.uni-lj.si (P.T.)
* Correspondence: mocehops@yandex.ru or a.lebedev@org.chem.msu.ru

Mass spectra and tentatively assigned structures of the secondary products of limonene aqueous chlorination

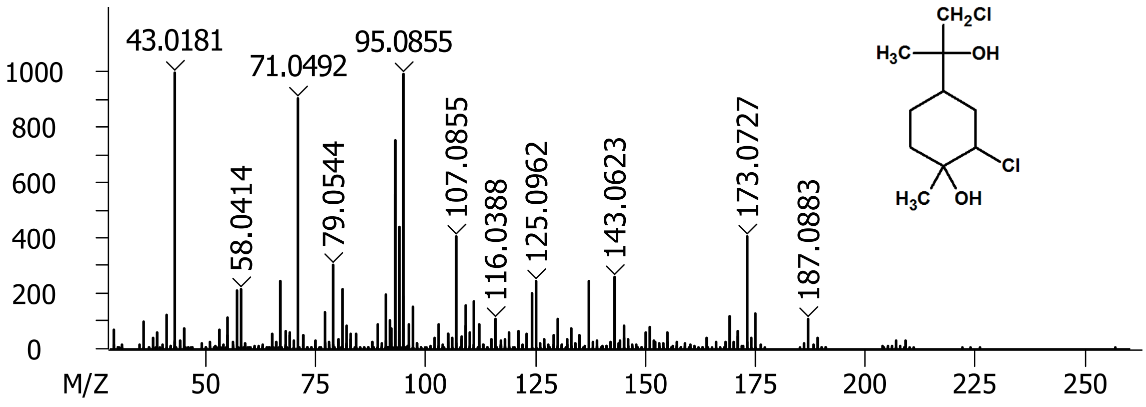

Figure S1. Mass spectrum and formula of 1-methyl-2-chloro-4(2-chloro-1-hydrohypropyl-2)cyclohexanol with RT 1104 s.

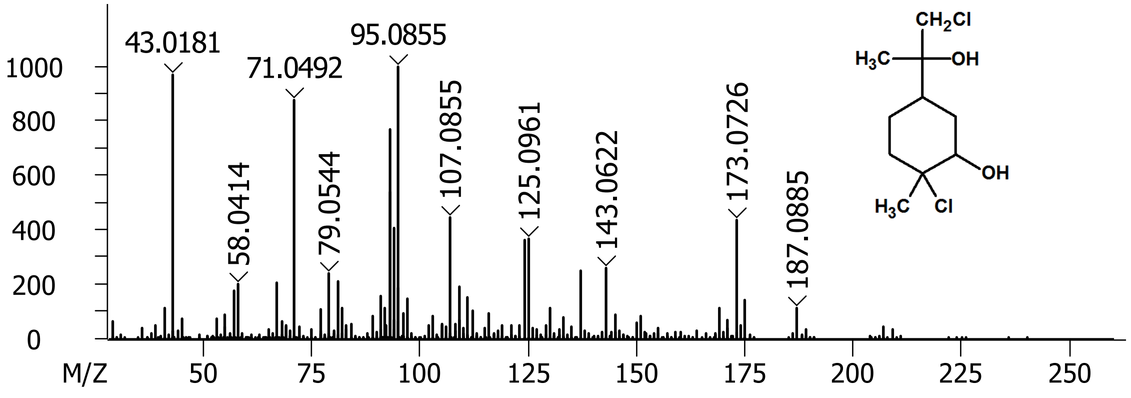

Figure S2. Mass spectrum and formula of 2-chloro-2-methyl-4(2-chloro-1-hydrohypropyl-2)cyclohexanol with RT 1107 s.

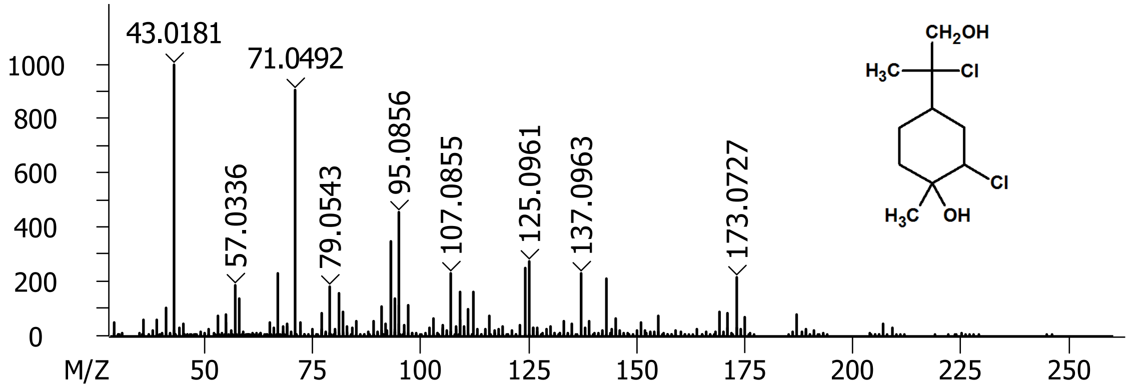

Figure S3. Mass spectrum and formula of 1-methyl-2-chloro-4(1-chloro2-hydroxy-propyl-2)cyclohexanol with RT 1112 s.

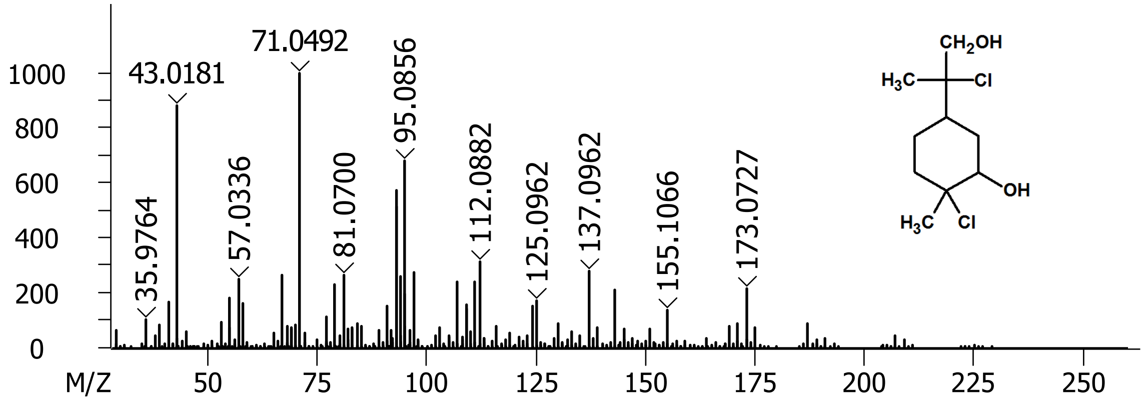

Figure S4. Mass spectrum and formula of 2-chloro-2-methyl-4-(1-chloro2-hydroxy-propyl-2)cyclohexanol with RT 1115 s.
